# Neuronal Circuits Underlying Apathy in Alzheimer's Disease

**DOI:** 10.1002/alz70857_102015

**Published:** 2025-12-24

**Authors:** Guang‐Wei Zhang

**Affiliations:** ^1^ University of Southern California, Los Angeles, CA, USA

## Abstract

**Background:**

Apathy is the most common neuropsychiatric symptom in Alzheimer's disease (AD), profoundly affecting patients and caregivers. Despite its prevalence, the neuronal mechanisms underlying apathy remain poorly understood. The septum, a limbic structure crucial for motivation and reward processing, exhibits early pathological changes in AD, suggesting its involvement in apathy‐related behaviors.

**Method:**

Apathy‐like behaviors were assessed in 5xFAD mice, a model of AD, across multiple age points (1, 2, 3, 6, and 9 months). Social interaction and motivation‐related behaviors were quantified using automated behavioral analysis tools. Electrophysiological recordings of septal neurons were performed, and optogenetic and chemogenetic manipulations were used to determine the causal role of GABAergic neurons in apathy‐related phenotypes.

**Result:**

5xFAD mice displayed early‐onset apathy‐like behaviors, including reduced social engagement and motivation, correlating with altered neuronal activity in the septum. Functional impairments in GABAergic neurons were identified, and targeted neuromodulation of these neurons rescued apathy‐like behaviors in the mouse model.

**Conclusion:**

These findings highlight the septum as a critical component in the circuitry underlying apathy in AD. Understanding septal dysfunction offers new opportunities for developing therapeutic strategies to address this debilitating symptom in AD.

